# Whole-Genome Restriction Mapping by “Subhaploid”-Based RAD Sequencing: An Efficient and Flexible Approach for Physical Mapping and Genome Scaffolding

**DOI:** 10.1534/genetics.117.200303

**Published:** 2017-05-02

**Authors:** Jinzhuang Dou, Huaiqian Dou, Chuang Mu, Lingling Zhang, Yangping Li, Jia Wang, Tianqi Li, Yuli Li, Xiaoli Hu, Shi Wang, Zhenmin Bao

**Affiliations:** * Key Laboratory of Marine Genetics and Breeding, Ministry of Education, Ocean University of China, Qingdao 266003, China; † Laboratory for Marine Fisheries Science and Food Production Processes, Qingdao National Laboratory for Marine Science and Technology, 266237, China; ‡ Laboratory for Marine Biology and Biotechnology, Qingdao National Laboratory for Marine Science and Technology, 266237, China

**Keywords:** *in vitro* linkage mapping, restriction map, RAD sequencing, genome scaffolding

## Abstract

Assembly of complex genomes using short reads remains a major challenge, which usually yields highly fragmented assemblies. Generation of ultradense linkage maps is promising for anchoring such assemblies, but traditional linkage mapping methods are hindered by the infrequency and unevenness of meiotic recombination that limit attainable map resolution. Here we develop a sequencing-based “*in vitro*” linkage mapping approach (called RadMap), where chromosome breakage and segregation are realized by generating hundreds of “subhaploid” fosmid/bacterial-artificial-chromosome clone pools, and by restriction site-associated DNA sequencing of these clone pools to produce an ultradense whole-genome restriction map to facilitate genome scaffolding. A bootstrap-based minimum spanning tree algorithm is developed for grouping and ordering of genome-wide markers and is implemented in a user-friendly, integrated software package (AMMO). We perform extensive analyses to validate the power and accuracy of our approach in the model plant *Arabidopsis thaliana* and human. We also demonstrate the utility of RadMap for enhancing the contiguity of a variety of whole-genome shotgun assemblies generated using either short Illumina reads (300 bp) or long PacBio reads (6–14 kb), with up to 15-fold improvement of N50 (∼816 kb-3.7 Mb) and high scaffolding accuracy (98.1–98.5%). RadMap outperforms BioNano and Hi-C when input assembly is highly fragmented (contig N50 = 54 kb). RadMap can capture wide-range contiguity information and provide an efficient and flexible tool for high-resolution physical mapping and scaffolding of highly fragmented assemblies.

NEXT-GENERATION sequencing (NGS) technologies, which enable the simultaneous production of billions of short reads at a very low cost per sequenced base, have provided unprecedented possibilities for genome sequencing (Shendure and Ji 2008; van Dijk *et al.* 2014; Goodwin *et al.* 2016). The recent advances in sequencing throughput and genome-assembly algorithms have resulted in a rapid proliferation of whole-genome shotgun (WGS) assemblies that represent the cornerstones for decoding genome structures and functions in a wide range of organisms (Ellegren 2014**)**. Despite these significant advances, many of the produced genome assemblies are highly fragmented, and one major obstacle in using WGS assemblies for important research applications such as genome-wide association or comparative genomics has been the lack of chromosomal positioning and contextualization of short sequence contigs (Alkan *et al.* 2011; Mascher and Stein 2014). Currently, challenges remain in generating well-assembled reference genomes due to the short reads produced via the NGS platforms and to the complexities of large eukaryotic genomes with high levels of repetitive elements (Earl *et al.* 2011; Treangen and Salzberg 2012; Goodwin *et al.* 2016).

Diverse strategies have been developed to boost the contiguity of WGS assemblies from short reads, with primary efforts on either increasing the read length generated from sequencing platforms or increasing the DNA fragment size used for sequencing. However, none of these strategies offers the “perfect” solution and each has its own strength and weakness. Long-read sequencing technologies can provide 10–15-kbp reads to build larger contiguous sequences but these are at the expense of high sequencing costs and/or high error rates (*e.g.*, PacBio SMRT or Oxford Nanopore) (Koren *et al.* 2012; Voskoboynik *et al.* 2013; Goodwin *et al.* 2015). Mate-pair libraries can be prepared from large-insert fosmid clones (35–40 kb), but remain technically challenging as custom-modified vectors are required and a complicated experimental procedure is involved (Williams *et al.* 2012; Wu *et al.* 2012). The “subhaploid”-based sequencing methods [*e.g.*, fosmid dilution pool sequencing, contiguity-preserving transposition sequencing (CPT-seq)] provide useful midrange contiguity information, but rely on extensive sequencing of hundreds to thousands of subhaploid pools (Kitzman *et al.* 2011; Zhang *et al.* 2012; Adey *et al.* 2014). The Hi-C and related methods can capture long-range chromatin interactions at multimegabase-length scales (Burton *et al.* 2013; Kaplan and Dekker 2013; Putnam *et al.* 2016) but their performance is usually suboptimal on input assemblies with short scaffold sizes (Adey *et al.* 2014).

Physical maps are indispensable tools in early eukaryotic genome projects where they provide an essential framework for ordering and joining sequence data, genetically mapped markers, and large-insert clones, and can also be used alone to isolate genes of interest, to home in on particular regions for sequencing, or to compare the organizations of different species’ genomes (Meyers *et al.* 2004; Lewin *et al.* 2009; van Oeveren *et al.* 2011). Despite these advantages, traditional physical mapping approaches have been, however, less favorable in the NGS era, because the creation and profiling of bacterial-artificial-chromosome (BAC) libraries remains labor intensive, time consuming, and expensive; *e.g.*, physical mapping based on a 10× human BAC library would have to deal with ∼200,000 BAC clones. As an appealing alternative to clone-based physical mapping approaches, optical mapping generates genome-wide restriction maps based on sizing of restriction fragments (Teague *et al.* 2010; Neely *et al.* 2011), which can provide both midrange and long-range contiguity information and has been widely applied in finishing large, complex eukaryotic genomes (Zhou *et al.* 2009; Lam *et al.* 2012; Dong *et al.* 2013). Nevertheless, efficient and accurate application of this methodology relies on expensive instruments (*e.g.*, OpGen Argus and BioNano Genomics Irys systems) (Levy-Sakin and Ebenstein 2013), limiting its accessibility to ordinary laboratories.

Physical ordering of genome-wide markers represents an appealing way to generate high-resolution physical maps in a rapid and cost-effective manner. The HAPPY mapping method, which was invented by Dear and Cook (1993), holds great promise to achieve this goal (Piper *et al.* 1998; Konfortov *et al.* 2000; Hall *et al.* 2002; Eichinger *et al.* 2005). HAPPY mapping is a genome mapping method that is analogous to classical linkage mapping except that the chromosome breakage and segregation are replaced by *in-vitro* analogs. Its procedure involves breaking intact genomic DNA at random, segregating the fragments into aliquots (∼0.3–0.7 haploid genome equivalent; Dear 2005) by limiting dilution and measuring the frequency of cosegregation of markers among the aliquots. It generates a map based on the premise that linked pairs of markers will cosegregate significantly more frequently than unlinked markers and in a manner that is proportional to their physical proximity (Dear and Cook 1993). Unlike classical linkage mapping methods, HAPPY mapping does not require any polymorphic markers; so any piece of DNA can be mapped to a genomic region. HAPPY mapping can also be easily adapted to any desired level of resolution, in particular, to a high resolution of genome maps (Konfortov *et al.* 2000), in contrast to limited resolution of classical linkage mapping methods due to the infrequency of meiotic recombination (*e.g.*, for human, one marker per 10^6^ bp; White and Lalouel 1988). However, because each aliquot in a HAPPY panel contains very little genomic DNA (*e.g.*, <3 pg for human DNA; Dear 2005), the lack of faithful amplification of each aliquot DNA to provide enough material for genotyping a large number of markers has been the bottleneck of this method (Jiang *et al.* 2009), and this has prevented such a simple and powerful method from coming into general use since it was invented 20 years ago.

Here, we develop a new approach by incorporating two major technical improvements to the original HAPPY mapping method: (i) use of fosmid/BAC clone pools as a HAPPY mapping panel to bypass the requirement of PCR-based amplification of each aliquot of DNA, and (ii) use of the 2b-restriction site-associated DNA sequencing (2bRAD-seq) (Wang *et al.* 2012) for high-throughput marker profiling and whole-genome restriction mapping. The new approach, which we called RadMap, provides an efficient and flexible method for high-resolution physical mapping and genome scaffolding. We perform extensive analyses (both *in silico* and experimentally) to validate the power and accuracy of our approach in human and the model plant *Arabidopsis thaliana* by generating high-quality restriction maps and enhancing the contiguity of various WGS assemblies generated using either short Illumina paired-end reads (300 bp) or long PacBio reads (6–14 kb).

## Materials and Methods

### *In silico* data sets

Four simulation data sets were created from the genome of the model plant *A. thaliana* (∼120 Mbp, five chromosomes, version TAIR10) and human genome (∼3.1 Gbp, 22 autosomes + *X* + *Y*, version GRCh37), by *in-silico* generation and sequencing of 100 fosmid (40-kb) and 100 BAC (100-kb) clone pools for each species. Briefly, DNA fragments with a mean size of 40 kb (fosmid) and 100 kb (BAC) were randomly sampled from the reference genome sequence of each species. These fragments were then distributed into 100 clone pools evenly, with each pool covering ∼0.5× haploid genome. 2bRAD-seq was simulated by extracting all *Bsa*XI tags from each pool, constituting the final mapping data sets.

### Preparation of the fosmid mapping panel

Fosmid library pool construction for *A. thaliana* was performed by basically following the experimental procedure as previously described (Kitzman *et al.* 2011). Briefly, high molecular weight (HMW) genomic DNA was extracted from leaf tissues using the conventional cetyltrimethyl ammonium bromide method. The DNA was then end repaired and size selected as 30- to 48-kb fragments using field inversion agarose gel electrophoresis (FIGE; Bio-Rad, Hercules, CA). Ligation of the collected fragments into the fosmid vector pCC1FOS was according to the manufacturer’s protocols (CopyControl Fosmid Library Production kit; Epicentre). Fosmid clones were packaged using MaxPlaxi Lambda Packaging Extract. After bulk infection, the library was split into 164 pools with each consisting of ∼1000 clones (∼0.3× haploid genome coverage). Clone DNA was extracted from each pool using the FosmidMAX DNA Purification kit (Epicentre).

### 2bRAD-seq and genotyping

2bRAD libraries were constructed for each clone pool by using the type IIB restriction enzyme *Bsa*XI, and following the protocol developed by Wang *et al.* (2012). The adaptors with 5′-NNN-3′ overhangs were used to target all *Bsa*XI fragments in the *A. thaliana* genome. A unique barcode was incorporated into each library during library preparation, and then all libraries were pooled for single-end sequencing (1 × 50 bp) using an Illumina HiSeq2000 sequencer.

Raw reads were first preprocessed to remove unreliable ones with no restriction site, ambiguous basecalls (N), long homopolymer regions, or excessive low-quality positions using the RADtyping program under default parameters (Fu *et al.* 2013). The obtained high-quality reads were further filtered by mapping against ∼10× MiSeq PE300 WGS data set (generated in this study) using the SOAP2 program (parameters -M 4, -v 2, -p 1 -r 2; Li *et al.* 2009) to remove non-*Arabidopsis* reads (*e.g.*, derived from fosmid vector and host bacteria). Clean reads from all clone pools were combined together and assembled into “locus” clusters using the Ustacks program (Catchen *et al.* 2011) by allowing at most two mismatches (parameters -m 2 -M 4). A collection of consensus sequences from all locus clusters comprises a set of representative reference sites. For marker genotyping, supposing that the cluster depth of the *i*th site in the *j*th pool is *C_ij_*, this site is genotyped as “1” if *C_ij_* > *c* (*c* is set to 2 here), “0” otherwise. Only representative sites with the percentage of “presence” among all pools falling into the interval [*α*, 1-*α*] (*α* is set to 0.1 here) were retained for further map construction.

### Grouping and ordering of restriction sites

We denote n as the total number of markers and $D_{ij}$ as the Hamming distance of a pair of markers (*l_i_* and *l_j_*). If the physical distance between the two markers is very large (typically larger than DNA clone size), we have the expectation of $D_{ij}$ as $E\left(D_{ij}\right)=0.5n$ and $P\left(D_{ij}<\delta \right)<e^{-2\left(0.5n-\delta \right)/n},$ where δ<0.5n; the detailed proof can be found in Wu *et al.* (2008). To eliminate the effect of genotype uncertainties on the accuracy of map order, we develop a bootstrap-based minimum spanning tree (bMST) algorithm for grouping and ordering the markers.

Our iteration procedure is described as follows. Setting the initial iteration s=0, we subsample 80% of samples from the original data set and obtain the corresponding Hamming distance $D_{ij}^{s}$ for each pair of markers. We perform R replicates and obtain the distance vector for each pair of markers (*l_i_* and *l_j_*) as $\left\{D_{ij}^{s0},D_{ij}^{s1},\ldots ,D_{ij}^{sR}\right\}$. The pair of markers will be assigned into one group if there are at least 0.6*R* replicates satisfying $D_{ij}^{sr}<0.5n\left\{1+\text{ln}\left[p^{\left(s\right)}\right]\right\},$ where $p^{\left(s\right)}$ is chosen from $10^{-2}$ to $10^{-20}$ to produce about *n*/100 initial groups (*n* = total number of markers). Applying this rule to all the pairs of markers, we can obtain the initial linkage groups $\left\{L_{1}^{s},L_{2}^{s},\ldots ,L_{sk}^{s}\right\}.$. The minimum spanning tree (MST) algorithm (Wu *et al.* 2008) is used to determine an optimal order of markers within each linkage group.

In the second phase, we set the iteration s=s+1 and regard the above-determined linkage groups as *k* nodes, and define the distance between the nodes i and j as $d_{ij}^{s}=\text{min}\left\{D_{ab}^{s0}\;|\;a\in L_{i}^{s},\;b\in L_{j}^{s}\right\}.$. Similarity, we also perform R replicates by subsampling 80% of samples and obtain the distance vector for each pair of groups (*i* and *j*) as $\left\{d_{ij}^{s0},d_{ij}^{s1},\ldots ,d_{ij}^{sR}\right\}.$. The pair of groups will be clustered into the supergroup if there are at least 0.6*R* replicates satisfying $d_{ij}^{sr}<0.5n\left\{1+\text{ln}\left[p^{\left(s\right)}\right]\right\}.$. In this way, we can get the corresponding supergroups $\left\{L_{1}^{s},L_{2}^{s},\ldots ,L_{sk}^{s}\right\}.$ The MST algorithm is used again to determine an optimal order of markers within each supergroup. Then, the process of the second phase is repeated and stops when there is no change in the number of groups or the number of groups is smaller than the threshold defined by the user.

We have developed a user-friendly, integrated software package (AMMO) for implementing the marker grouping and ordering algorithms (Supplemental Material, File S1), the newest version of which is freely available at http://www2.ouc.edu.cn/mollusk/detailen.asp?id=752.

### Determination of the optimal sequencing coverage for RadMap

The choice of sequencing coverage is critical because proper sequencing coverage saves the total sequencing cost but without excessive false genotyping negatives. To determine the optimal sequencing coverage for RadMap, we conducted a mathematical analysis of the relationship of sequencing coverage and marker false negative rate (FNR).

Denote the average sequencing coverage as *C*. For a 2bRAD data set, the read depth *k* for each unique restriction site theoretically follows the Poisson distribution, assuming that all sites across the genome are evenly sequenced (Dou *et al.* 2012):

$$\text{Poisson}\left(k|C\right)∼\frac{C^{k}e^{-C}}{k!}.$$

If the observed depth of one site is no more than 2, we regard the genotype of this site as 0 or “undetermined,” indicating that this marker information cannot be captured using the threshold method. Therefore, the FNR can be represented as:

$$\text{FNR}=\underset{k<3}{\sum }\frac{C^{k}e^{-C}}{k!}=e^{-C}\left(1+C+0.5C^{2}\right).$$

### Input WGS assemblies and RadMap-based scaffolding

Shotgun genomic libraries were prepared for the ecotype Columbia (Col-0) of *A. thaliana*, and were sequenced based on Illumina MiSeq and PacBio RSII platforms. One paired-end DNA library with insert size of ∼500–550 bp was constructed by following the Illumina standard DNA library preparation protocol and was then sequenced using the Illumina MiSeq PE300 platform. Raw reads were first filtered to remove low-quality reads resulting from base-calling duplications or adapter contamination. Clean reads were assembled using four *de novo* assemblers: Celera (Myers *et al.* 2000), SOAPdenovo2 (Luo *et al.* 2012), ABySS (Simpson *et al.* 2009), and SPAdes (Bankevich *et al.* 2012). PacBio library preparation and sequencing was performed at the Yale Center for Genome Analysis (read length: 5–8 kb). PacBio reads were first preprocessed and error corrected based on Illumina PE300 reads using ECTools pipeline (Lee *et al.* 2014), and then they were assembled into contigs using a simple greedy algorithm implemented in IPython and Python (http://nbviewer.ipython.org/urls/raw.github.com/cschin/Write_A_Genome_Assembler_With_IPython/master/Write_An_Assembler.ipynb). The PacBio assembly data set (from a 20-kb insert library) recently released for the ecotype Ler-0 of *A. thaliana* was also included in our analysis, which was retrieved from the Pacific Biosciences Web site (http://www.pacb.com/blog/new-data-release-arabidopsis-assembly/).

The obtained WGS assemblies were used as the input for RadMap-aided scaffolding based on the hierarchical assembly algorithm. The RadMap-based scaffolding approach is analogous to the marker grouping and ordering method as described above except that the *Bsa*XI tags derived from the same contig were preassigned into one linkage group (*i.e.*, $\left\{L_{1}^{0},L_{2}^{0},\cdots L_{0k}^{0}\right\}$ are the contigs consisting of at least one *Bsa*XI tag). The effectiveness of RadMap in scaffolding WGS assemblies was evaluated using a series of metrics including linkage group number, N50 size, N90 size, genome coverage, and average map accuracy. The N50/N90 size is a weighted median statistic such that 50 or 90% of the entire assembly is contained in contigs or scaffolds equal to or larger than this value. Genome coverage refers to the percentage of the genome that is contained in the assembly. The contigs that are not anchored by scaffolding technologies are not included for calculation of genome coverage. The average map accuracy was measured using Kendall’s statistic and a detailed description about Kendall’s statistic can be seen in Wu *et al.* (2008). The gap sizes between contigs or scaffolds were estimated using the piecewise cubic Hermite interpolation method. We extracted and compared the physical distance and corresponding map distance of pairs of *Bsa*XI tags that were located in the same contigs, and used this information for model training and parameter estimation.

### Genome scaffolding of *A. thaliana* by BioNano and Hi-C

Scaffolding the WGS assemblies of *A. thaliana* by BioNano (optical mapping) and Hi-C was conducted for comparison with RadMap. For the BioNano technique, an assembled optical map (total size, 124.0 Mb; N50, ∼1.86 Mb; 101 contigs) of *A. thaliana* Col-0 generated by a previous study (Kawakatsu *et al.* 2016) was downloaded from http://signal.salk.edu/opticalmaps/Col-0.cmap. This physical map was generated from HMW DNA nicked with the Nt.BspQI enzyme using the IrysView platform (BioNano Genomics). The WGS assemblies of *A. thaliana* derived from short Illumina reads or long PacBio reads were converted into .cmap format and then aligned to the Col-0 optical map using the perl script (stitch.pl; Shelton *et al.* 2015) with default parameters (-e BspQI -f_con 20 -f_algn 40 -s_con 15 -s_algn 90 -n 20000 -T 1e-8). For the Hi-C technique, we retrieved the Hi-C data [National Center for Biotechnology Information Sequence Read Archive (SRA) accession number SRP043612] of *A. thaliana* Col-0 from a previous study (Feng *et al.* 2014), which were sequenced on an Illumina HiSeq2000 platform with pair-end 50-/51-nt reads. A total of 43.8 million Hi-C reads were aligned to the WGS assemblies of *A. thaliana* (∼94.7% mapping rate, ∼1700× coverage), which were then used to construct the scaffold graph and later to orient and order contigs using the tool SALSA (Ghurye *et al.* 2016) with the default settings (link score >0; at least five links for a pair of contigs). For technical comparison of RadMap with BioNano or Hi-C, we focus on three metrics: genome coverage, N50 and N90 of contig size, and scaffolding accuracy.

### Data availability

The sequencing data generated by this study were archived in the SRA database (2bRAD data, SRP068747; Illumina MiSeq and PacBio WGS data, SRP068748 and SRP068751). Simulated data and codes were included in the supplementary AMMO software package.

## Results

### Overview of the RadMap approach

**Figure 1:**
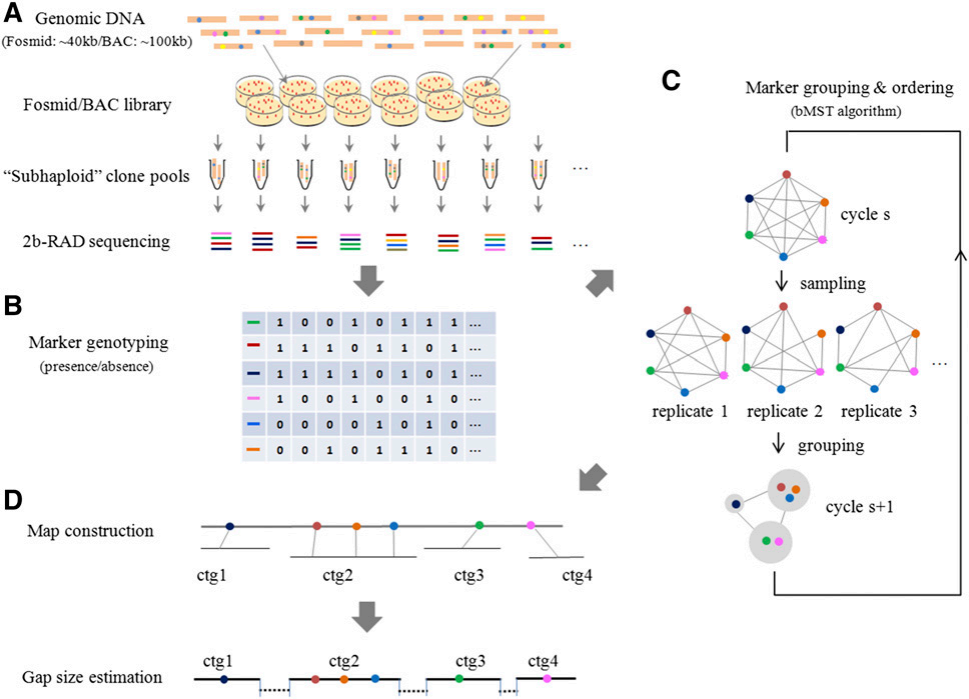
Overview of the RadMap approach for restriction mapping and genome scaffolding. (A) Generation and sequencing of subhaploid clone pools. A mapping panel is created by generating a large-insert fosmid (40 kb) or BAC (100 kb) library and then splitting it into hundreds of clone pools, with each pool representing less than one haploid genome (∼0.3–0.7×). 2bRAD libraries are prepared for each pool and then pooled together for high-throughput sequencing. (B) Marker genotyping. The presence (1) or absence (0) of a marker in each pool is determined according to the sequencing depth of the marker, and the coexisting frequencies of pairs of markers across clone pools are used to estimate the pair-wise distances between markers. (C) Marker grouping and ordering. A bMST algorithm is developed for grouping and ordering genome-wide markers. For each iteration, a certain number of clone pools are randomly picked up to estimate the pair-wise distance between markers, and then markers are assigned into different groups according to a specified threshold. One pair of markers will be placed together if they exist in one group for >60% of replications. A new cycle starts by regarding the groups generated from former cycle as new nodes, and the pair-wise distance between groups defined as the minimal distance among tags mapped along them. (D) Genome scaffolding and gap-size estimation. For scaffolding a WGS-based preassembly, the bMST algorithm can take the contigs/scaffolds from the assembly as the input for grouping and ordering as long as each contig/scaffold contains at least one *Bsa*XI tag. The gap size between anchored contigs/scaffolds can be estimated based on a linear regression model established by comparing the map distance and true physical distance between markers. ctg, contig.

The schematic illustration of the RadMap approach is shown in Figure 1. Briefly, a mapping panel is created by generating hundreds of large-insert fosmid/BAC clone pools, with each pool covering less than one haploid genome (*e.g.*, ∼0.3–0.7×; Figure 1A). Unlike other clone-based physical mapping approaches [*e.g.*, whole-genome profiling (WGP), van Oeveren *et al.* 2011; BAC-HAPPY (BAP), Vu *et al.* 2010], our clone pooling approach (see *Materials and Methods* for details) does not require isolation and maintenance of individual fosmid/BAC clones in multiwell plates, the procedure of which can be labor intensive, time consuming, and expensive. The cloned DNA isolated from each pool is subject to 2bRAD-seq and genotyping, with each marker genotyped based on its presence or “absence” in a given pool (Figure 1B). The genotypes collected from all pools are used to estimate the pair-wise distance between markers. To determine an optimal marker order, we develop a bMST algorithm for the grouping and ordering of genome-wide markers (Figure 1C; see *Materials and Methods* for algorithm details), which is well suited for dealing with noisy or incomplete mapping data. For genome scaffolding, contigs/scaffolds from preassemblies can be directly anchored with the aid of the constructed map or similarly ordered by regarding contigs/scaffolds as nodes (Figure 1D). The gap size between anchored contigs/scaffolds can be estimated based on a linear regression model that is established by comparing the map distance and true physical distance between markers. To facilitate the research community to implement our approach, a user-friendly, integrated software package (AMMO) is developed for whole-genome restriction mapping and genome scaffolding (File S1).

### *In silico* analysis

Simulation-based evaluation of the RadMap approach was first performed to investigate the methodological feasibility and optimal mapping parameters. Four simulation data sets were created from the entire *A*. *thaliana* genome (five chromosomes) and human genome (22 autosomes + *X* + *Y*) by generating 100 fosmid (40-kb) and 100 BAC (100-kb) clone pools for each species for *in silico* 2bRAD-seq and genotyping (*Bsa*XI enzyme used here). A total of 35,618 unique *Bsa*XI tags (*i.e.*, markers) were successfully typed for *A*. *thaliana*, whereas 1,118,736 unique *Bsa*XI tags were typed for human, with average marker distances of 3.3 and 3.0 kb, respectively (Table S1 in File S2). For the *Arabidopsis* 40-kb data set, the 35,618 markers fell into 40 linkage groups, while the linkage groups further reduced to 20 when the cloned fragment size increased to 100 kb (Table 1). The performance of RadMap is also prominent for human mapping panels with high marker density, where 407 and 361 linkage groups were obtained for the 40- and 100-kb data sets, respectively. The map continuity and accuracy statistics during the iterative marker-grouping process are detailed in Table S2 in File S2. As expected, many initially produced groups were comprised of relatively fewer markers with few grouping errors, and further grouping led to progressively larger groups with few mis-assemblies introduced. Only slight changes in group numbers were observed after the ninth iteration. The final N50 sizes of obtained restriction maps for *Arabidopsis*-40 kb, *Arabidopsis*-100 kb, human-40 kb, and human-100 kb were 4.1, 12.7, 9.7, and 11.0 Mb, respectively (Table 1). Remarkably, the average accuracy of within-group marker orders as measured by Kendall’s metrics was >99.7% for all cases (Figure S1 in File S2 and Table 1). Optimal sequencing coverage analysis suggests that the average sequencing coverage of RadMap should be no less than 7× to ensure a low FNR (< 0.05) of marker genotyping (Figure S2 in File S2); but in practice, higher sequencing coverage (*e.g.*, 10–20×) may be preferred to account for noisy practical data. Overall, our simulation analysis supports that the RadMap approach coupled with the hierarchical mapping algorithm allows for the effective build of high-quality restriction maps even in species with large and complex genomes.

**Table 1:** Summary of RadMap restriction mapping based on simulation data sets

	*A. thaliana*		*H. sapiens*	
Clone size (kb)	40	100	40	100
No. of linkage groups	40	20	407	361
N50 (Mb)*^a^*	4.1	12.7	9.7	11.0
N90 (Mb)*^a^*	0.8	4.9	2.7	2.8
Coverage (%)*^b^*	98.4	98.5	99.2	99.4
Accuracy (%)*^c^*	99.7	99.9	99.9	99.9

a The N50 of a map is defined as the length *N* for which 50% of the entire map is contained in linkage groups with lengths equal to or larger than *N*. The map N90 is similarly defined.

b Genome/chromosome coverage refers to the percentage of the genome/chromosome that is contained in the map.

c Calculated according to Kendall’s statistic.

### Restriction mapping based on real data sets

**Figure 2:**
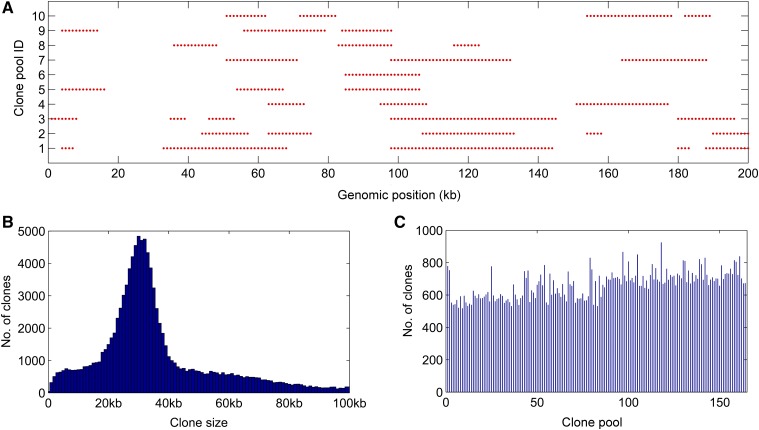
Generation and sequencing of 164 subhaploid clone pools in *A. thaliana*. (A) Visualization of fosmid clones distributed along the reference genome. A partial region of chromosome *1* (0–1 Mb) is chosen for display of 10 clone pools. One red ● represents a *Bsa*XI tag. (B) The histogram of estimated insert sizes of fosmid clones. It is shown that ∼65% of clones fall into the range of 20–40 kb. (C) Distribution of inferred clone numbers across all clone pools. The average number of clones per pools is 665 (representing 0.22× haploid genome), with an SD of 85.

To evaluate the performance of RadMap on real data sets, we generated and sequenced 164 fosmid clone pools from *A*. *thaliana*, with each pool targeting ∼0.3× haploid genome. A total of 1.5 billion 2bRAD reads were produced for the 164 clone pools, with an average sequencing depth of 47× per pool (Table S3 in File S2). To check the quality of obtained mapping data, we reconstructed fosmid clones in each pool by detecting the blocks of mapped tags along the reference genome (Figure 2A), based on the premise that the tags derived from the same clone will be positioned next to their correct neighbors, generating one cluster along the reference genome. The mean size of reconstructed clones was ∼35 kb (∼65% of clones in the range of 20–40 kb), using the average marker distance (3.4 kb) as the basis for clone size estimation (Figure 2B). Some reconstructed clones (28%) were longer than 40 kb, likely resulting from the emergence of overlapping clones during clone reconstruction. Each pool contained ∼665 reconstructed clones, representing 0.22× haploid genome (Figure 2C).

One of the challenges in *de novo* analyzing of real RadMap data is to reconstruct high-quality reference restriction sites from noisy sequencing data. In our approach, *Bsa*XI tags were first filtered by mapping against ∼10× WGS data (Figure S3 in File S2) to remove non-*Arabidopsis* tags (*e.g.*, derived from fosmid vector and host bacteria). The “clean” tags were then clustered, resulting in 34,753 reference *Bsa*XI sites. After eliminating those sites that gave fewer (<16) or no positive typing, or gave an unreasonably high proportion of positive typing (>148 positives of 164 pools); 32,378 reference sites ultimately remained. These reference sites represent 91% of the unique BsaXI sites in silico predicted from the A. thaliana genome, highlighting the effectiveness of our de novo site-reconstruction strategy. Marker linkage analysis first generated 1398 primary linkage groups, and after the ninth grouping step, the number of linkage groups shrunk in 1066 (∼24% reduction) with the overall accuracy of marker order being 97.8% (Table 2). The generated restriction map has an N50 size of 265 kb and covers ∼109 Mb (92%) of the *A. thaliana* genome, providing a valuable high-resolution physical map for genome scaffolding and other genomic applications.

**Table 2:** Summary of hierarchical restriction mapping for the real data set of *A. thaliana*

	No. of groups	No. of groups (>10 tags)
Step 1	1398	638
Step 2	1273	608
Step 3	1221	593
Step 4	1174	581
Step 5	1141	575
Step 6	1113	565
Step 7	1097	565
Step 8	1073	557
Step 9	1066	554

### Genome scaffolding by RadMap

**Figure 3:**
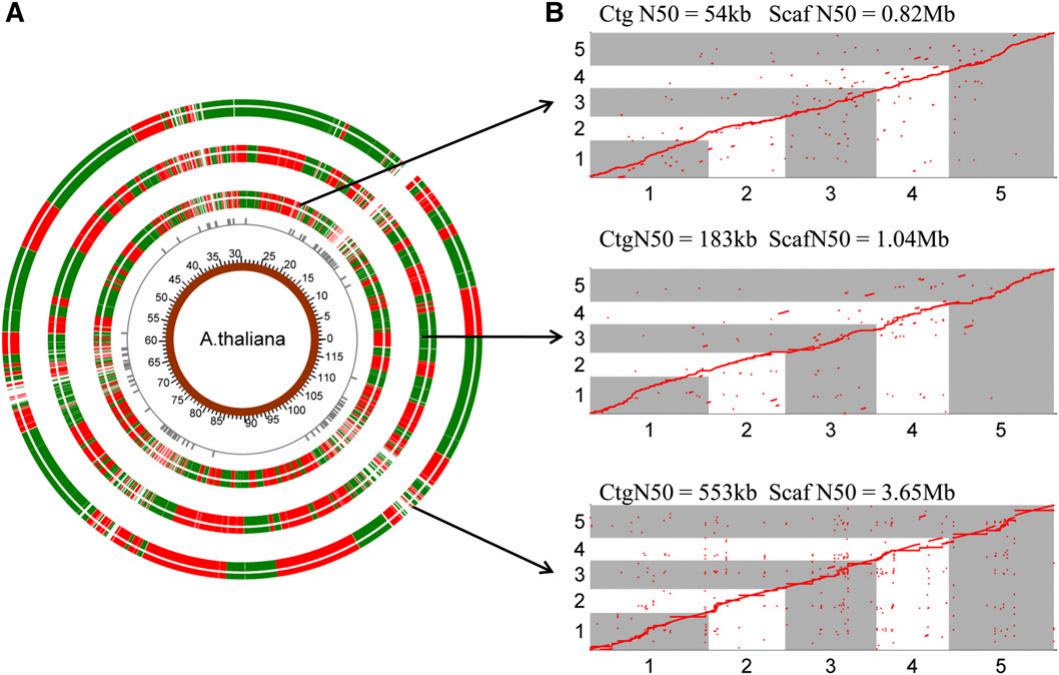
RadMap scaffolding of different WGS assemblies of *A. thaliana*. (A) Overview of three RadMap-based assemblies, with 15.1-, 5.7-, and 6.6-fold improvement of assembly contiguity. From inner to outer rings are genome coordinates, *Bsa*XI sites with between-site distances over 40 kb, and RadMap scaffolding of three WGS assemblies generated based on Illumina MiSeq PE300, PacBio-5 kb, and PacBio-14 kb data sets (Table 3), respectively. The junctions between the red and green bands for the outermost three rings represent the gaps in the assembled genome, and most gaps result from genomic regions containing very sparse *Bsa*XI sites (between-site distances >40 kb). (B) Dot-plot comparison of the RadMap-based assemblies and the reference genome (five chromosomes), showing high accuracy of contig linkage with Kendall’s statistic >0.98 (Table 3). One red ● represents one *Bsa*XI tag. Ctg, contig; Scaf, scaffold.

**Figure 4:**
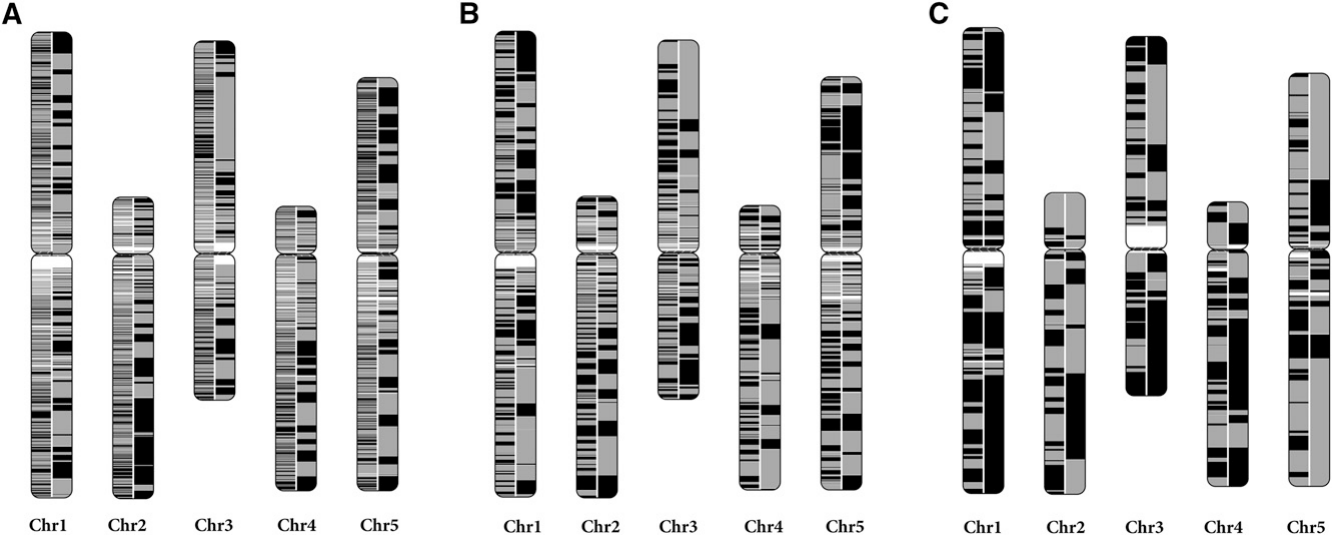
The continuity of the RadMap-based assemblies. The *A. thaliana* chromosomes are painted with assembled contigs. Alternating shades indicate adjacent contigs, and each vertical transition from gray to black represents a contig boundary or alignment breakpoint. The left half of each chromosome shows the input assembly of (A) 25× MiSeq PE300 data set, (B) 5× PacBio-5 kb data set, and (C) 5× PacBio-14 kb data set, while the right half shows the corresponding RadMap-based assembly. The RadMap-based assemblies are considerably more continuous, with 15-, 6-, and 7-fold improvement of N50 and 12-, 12-, and 5-fold improvement of N90. Chr, chromosome.

**Figure 5:**
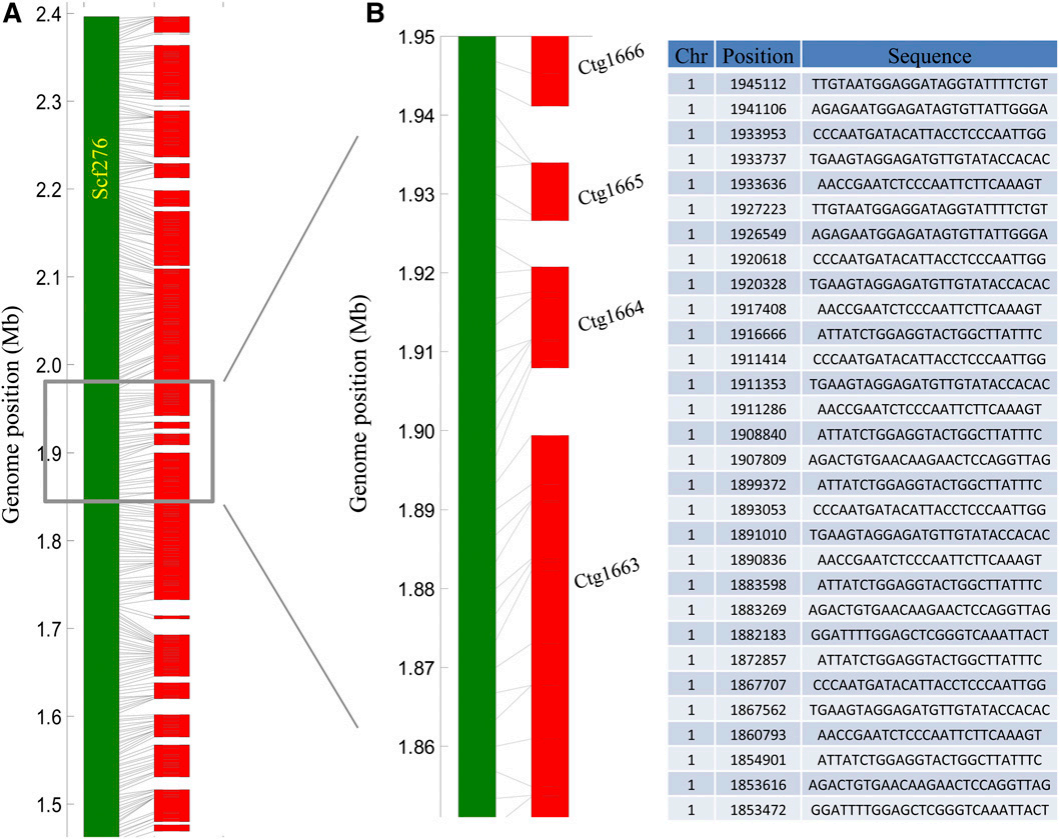
Examples of RadMap-linked contigs. (A) Overview and (B) zoomed-in detail of one genomic region located on the chromosome *1* (1.45–2.40 Mb) of *A. thaliana*, which consists of 20 contigs generated from 25× MiSeq PE300 data set, with *Bsa*XI tag or contig orders highly consistent with the reference genome. Chr, chromosome; Ctg, contig.

We then evaluated the performance of RadMap for genome scaffolding by generating a variety of WGS assemblies (Table S4 in File S2) for *A. thaliana* using either short Illumina reads (300 bp) or long PacBio reads (6–14 kb). For the Illumina MiSeq data set, ∼25× paired-end reads (PE300) were produced and used to create draft assemblies using four *de novo* assemblers: Celera (Myers *et al.* 2000), SOAPdenovo2 (Luo *et al.* 2012), ABySS (Simpson *et al.* 2009), and SPAdes (Bankevich *et al.* 2012). Among these assemblers, Celera generated the best assembly with the contig N50 of 54 kb and a total length of 116 Mb (Table S5 in File S2), which was chosen for further analysis. Our RadMap approach facilitated anchoring 3322 contigs with a genome coverage of 93% (Figure 3A). The final assembly contained 460 scaffolds with an N50 size of 0.82 Mb (Table 3), representing a 15-fold improvement of N50 from the initial assembly (Figure 4A). The consistency between the obtained marker order by RadMap and actual marker positions along the reference genome was shown in Figure 3B, where most scaffolds contained no significant translocation or inversion errors. The gap distribution for the final assembly was investigated, and most gaps were located in the regions with a relatively low density of *Bsa*XI tags (Figure 3A). Among all the 2877 contigs links, only 56 errors (*i.e.*, mis-joined contigs) were observed, indicating that 98.1% contigs were positioned next to the correct neighbors by RadMap. Figure 5A shows the alignment of a representative scaffold (scf276) to chromosome *1* of *A. thaliana* (1.45–2.40 Mb), with its zoomed-in details shown in Figure 5B. This scaffold consisted of 20 contigs, with no linkage error introduced during the RadMap-based scaffolding process.

**Table 3:** Metrics for scaffolding the WGS assemblies of *A. thaliana* by RadMap, BioNano, and Hi-C

*De novo* WGS assemblies	MiSeq	PacBio1	PacBio2
Read length (bp)	300	5000	14,000
Read depth	25×	5×	5×
Coverage	115.7 (97.2%)	121.7 (101.6%)	124.9 (104.3%)
No. of contigs	4502	2640	799
N50 size (kb)	54.1	183.4	552.7
N90 size (kb)	12.2	15.5	100.5
RadMap-based scaffolding			
Coverage	109.8 (91.7%)	115.9 (96.7%)	117.5 (98.1%)
No. of scaffolds	460	273	99
N50 size (kb)	815.9	1038.0	3,653.3
N90 size (kb)	144.5	191.9	481.7
No. of links	2877	620	268
No. of wrong links*^a^*	56	11	4
Accuracy (%)	98.1	98.2	98.5
BioNano-based scaffolding			
Coverage	36.1 (30.1%)	72.3 (60.4%)	75.2 (62.8%)
No. of scaffolds	34	56	35
N50 size (kb)	1433.3	1700.7	2,596.9
N90 size (kb)	572.9	685.2	1,164.7
No. of links	75	166	87
No. of wrong links*^a^*	0	0	0
Accuracy (%)	100	100	100
Hi-C-based scaffolding			
Coverage	105.1 (87.8%)	117.6 (98.2%)	120.3 (100.5%)
No. of scaffolds	618	126	62
N50 size (kb)	373.1	5441.1	20,739
N90 size (kb)	64.9	582.8	2,914
No. of links	2063	1413	439
No. of wrong links*^a^*	22	28	31
Accuracy (%)	98.9	98.0	92.9

a Wrong links refer to the cases where contigs are positioned next to the wrong neighbors when comparing to the reference genome.

We next sequenced a PacBio genomic library generated from the ecotype Col-0 of *A. thaliana*, with the average read length of 5.3 kb after error correction using Illumina short reads (Table S4 in File S2). After assembling these long reads at only 5× sequencing coverage, we were able to produce an assembly with the contig N50 of 183 kb (Table 3), which was three times better than the Illumina PE300-based assembly (Celera version: 54 kb). By using 2640 contigs as the input, RadMap assigned them into 273 scaffolds, with a 6-fold improvement of N50 (1.0 Mb) and 12-fold improvement of N90 (192 kb) (Figure 4B and Table 3). Among all the 620 links, only 11 wrong links were observed, indicating a high accuracy of 98.2% for RadMap in contig anchoring (Figure 3B and Table 3).

We also attempted to generate a chromosome-scale assembly for the ecotype Ler-0 of *A. thaliana* by coupling RadMap with the recently released long reads (∼14 kb) generated from the PacBio RSII system. The contig N50 obtained by assembling these long reads reached up to 553 kb when using only 5× sequencing reads. RadMap could efficiently anchor input contigs with the final assembly having an N50 size of 3.6 Mb, ∼6.6 times better than that of the initial assembly (Figure 4C and Table 3). Overall, our results suggest that RadMap can impressively boost the contiguity of *de novo* genome assemblies, especially for those that are highly fragmented.

**Figure 6:**
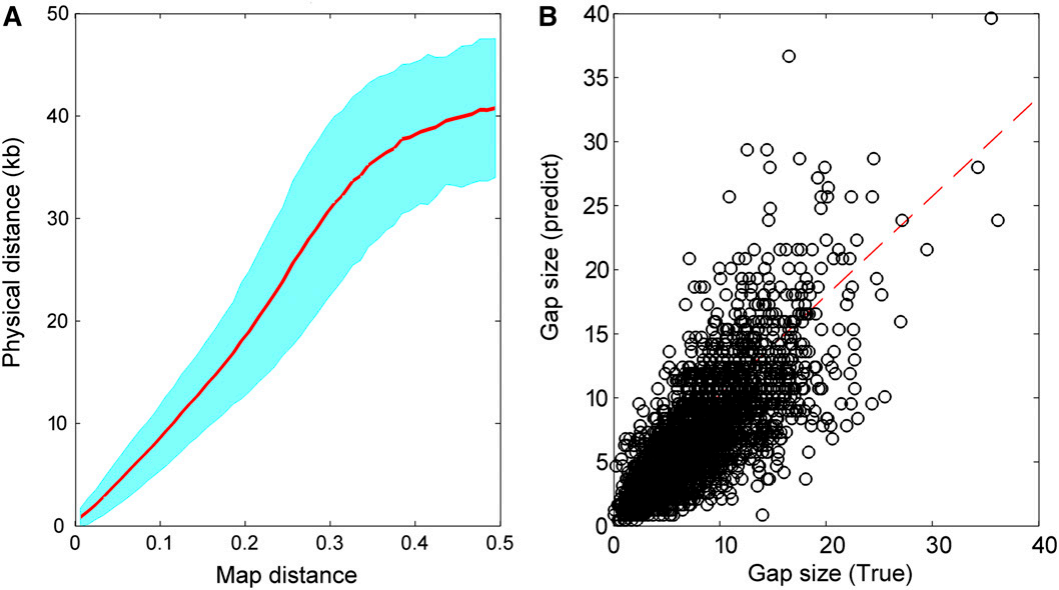
Gap-size estimation. (A) The relationship between map distance and true physical distance. The inter-contig map distances are obtained from the RadMap assembly generated using the MiSeq PE300 data set and the corresponding true physical distances are determined according to the reference genome. The map distances range from 0 to 0.5 and are split into 50 bins. The red line refers to the average physical distance of pairs of markers for each bin, and the cyan region denotes the corresponding SE region. Note only the pairs of markers with physical distance no longer than 50-kb apart are included here. (B) Comparison of the true and predicted inter-contig gap sizes. The dashed line indicates the linear least squares fit of *y* = 0.7752*x* + 2496, with the Pearson correlation *r* of 0.75.

As for gap-size estimation, we reasoned that the map distance between a pair of markers might enable the estimation of gap sizes between contigs/scaffolds. Figure 5A shows how map distance related to the known physical distances for 427,522 pairs of markers (<50 kb apart). Larger SE of physical distance could be observed with the increasing map distance, because a fosmid-range panel contains DNA fragments with a mean size of ∼40 kb, beyond which linkage information is not significantly above the background “noise” as seen between unlinked markers. We predicted the inter-contig gap sizes using the piecewise cubic Hermite interpolation method in which the model was trained using the data set from Figure 6A, with the corresponding comparison between the true and predicted gap sizes shown in Figure 6B. It can be fitted well using the linear regression model *y* = 0.7752*x* + 2496, with the Pearson correlation *r* of 0.75, indicating a reasonable estimation of gap sizes using this model.

### Scaffolding comparison of RadMap with BioNano and Hi-C

We further compared the scaffolding performance of RadMap with the two most widely used scaffolding methods, BioNano (Lam *et al.* 2012) and Hi-C (Burton *et al.* 2013). The BioNano and Hi-C data of *A. thaliana* Col-0 were retrieved from previous studies (Feng *et al.* 2014; Kawakatsu *et al.* 2016) and their scaffolding performance was evaluated based on the same set of input WGS assemblies that were used in RadMap evaluation. In terms of the completeness of scaffolded assemblies (*i.e.*, genome coverage), RadMap performs well (91.7–98.1%), and is comparable to Hi-C (87.8–100%), but much better than BioNano (30.1–62.8%) (Table 3). The poor performance of BioNano may be related to its relatively high mapping error rates (*e.g.*, fragment sizing errors, false cuts, and missing cuts; Li *et al.* 2016; Verzotto *et al.* 2016) and/or inefficiency of capturing small contigs that contain too few nicking sites to be useful for alignments. Further comparison of RadMap and Hi-C revealed that RadMap performs better than Hi-C in scaffolding the highly fragmented Illumina-based input assembly (input N50, 54 kb; N90, 12 kb). The N50 and N90 contiguity of the input Illumina assembly is remarkably improved by RadMap to 816 and 145 kb, respectively, in contrast to 373 and 65 kb in Hi-C (Table 3). For PacBio-based input assemblies, Hi-C is apparently advantageous over RadMap in producing a much longer N50 (5.4–20.7 Mb *vs.* 1.0–3.7 Mb in RadMap) and N90 (583–2914 kb *vs.* 192–482 kb in RadMap), though its scaffolding accuracy (92.9–98.0%) is slightly lower than RadMap (98.2–98.5%) (Table 3). Overall, our results suggest that RadMap would be most advantageous in scaffolding highly fragmented assemblies, which can be a common situation if initial assemblies are generated using short-read sequencing platforms.

## Discussion

### Technical barriers conquered by RadMap

The concept of HAPPY mapping, though very attractive, has not come into general use since its invention ∼20 years ago, due to the technical barriers imbedded in its original methodology (Dear and Cook 1993; Jiang *et al.* 2009). It has been envisioned as an “old-can-be-new-again” methodology in the NGS era (Jiang *et al.* 2009), which calls for the development of “next-generation” HAPPY mapping approach(es) based on NGS platforms. In this study, we developed the RadMap approach by incorporating technical improvements to the original HAPPY mapping method and by combining this with NGS platforms to provide an efficient and flexible way for high-resolution physical mapping and genome scaffolding.

#### Mapping panel:

In the RadMap approach, fosmid or BAC clone pools are used to constitute a mapping panel to bypass the requirement of *in-vitro* amplification of a trace amount of DNA (*e.g.*, the haploid DNA mass is ∼3 pg for human; Dear 2005) in the classic HAPPY mapping experiments, which is technical challenging and has been considered as the bottleneck of the original methodology (Jiang *et al.* 2009). Such “*in-vivo*” propagation of each fragment in bacteria ensures the unbiased “amplification” of the whole fragment, and is thus superior to the PCR- or multiple displacement amplification-based amplification approaches. In addition, fosmid and BAC libraries are relatively easy to establish (*e.g.*, commercial kits and services are available), and for many model species, such libraries already exist. Even for nonmodel species, creating new mapping panels is relatively easy and cost effective because our clone pooling approach does not involve the isolation and maintenance of individual fosmid/BAC clones.

#### Marker system:

Traditional HAPPY mapping experiments were mostly based on low-throughput, labor-intensive marker systems (*e.g.*, PCR-based STS markers; Dear 2005). The advent of NGS technologies has greatly stimulated the development of a variety of high-throughput genotyping-by-sequencing (GBS) methods that use restriction enzymes for genome complexity reduction to achieve genome-wide genotyping at minimal labor and cost (Andrews *et al.* 2016; Wang *et al.* 2017). Among available GBS methods, 2bRAD is more competent for coupling with the HAPPY mapping approach. First, 2bRAD can produce short uniform tags (32–36 bp) by using type IIB restriction enzymes to achieve an even sequencing depth across restriction sites (Wang *et al.* 2012), which serves as an important technical basis for reliable dominant marker genotyping (*i.e.*, tag presence *vs.* absence) as demonstrated in our previous studies (Fu *et al.* 2013; Jiao *et al.* 2014; Tian *et al.* 2015). Second, 2bRAD can potentially target all restriction sites in the genome of interest (Wang *et al.* 2012), and is thus well suited for generating a high-resolution physical map. Third, 2bRAD offers a very simple library preparation procedure, which only consists of four major steps (digestion, ligation, amplification, and barcoding) and can be completed in ∼4 hr (Wang *et al.* 2012). Lastly, 2bRAD library preparation and sequencing are very cost effective. With our recent technical improvements (*i.e.*, five tag concatenation for Illumina paired-end sequencing), the cost can reach ∼$8 per sample for library preparation and ∼$9 for sequencing 10 million reads per sample (Wang *et al.* 2016).

#### Marker ordering algorithm:

Searching for an optimal order of high-density markers is computationally challenging. Enumerating all the possible orders quickly becomes infeasible because the total number of distinct orders is proportional to *n*!, which can be very large even for a small number *n* of markers (Wu *et al.* 2008). To circumvent this problem, we developed a bMST algorithm for grouping and ordering of genome-wide markers. The MST algorithm efficiently determines the optimal order of markers by computing the MST of an associated graph, and has been previously shown to outperform other commonly used tools (*e.g.*, JOINMAP, CARTHAGENE, and RECORD) for construction of genetic linkage maps (Wu *et al.* 2008). Because HAPPY mapping data can be noisy or incomplete, our bMST algorithm groups and orders markers in a stepwise manner, and by combining MST with a bootstrap sampling strategy, the reliability of marker order is iteratively measured and controlled. Our bMST algorithm is therefore well suited for dealing with noisy or incomplete mapping data, and its effectiveness has been demonstrated in our simulation and real data-based analyses.

### Comparison of RadMap with other physical mapping/genome scaffolding methods

Physical maps provide an essential framework not only for ordering and joining sequence data, but also for detecting structural variations or comparing the genome organizations of different species (Lewin *et al.* 2009). RadMap can generate high-resolution physical maps in a rapid and cost-effective manner, with several advantages over existing physical mapping methods. A technical comparison between RadMap and other physical mapping methods is summarized in Table 4. First, RadMap and optical mapping can achieve high-resolution mapping by the construction of genome restriction maps, while other maps are usually of lower resolution due to the low-throughput marker system used in the traditional HAPPY map and radiation hybrid (RH) map (Cox *et al.* 1990) or the ordering of only large-insert clones but not markers (*i.e.*, tags within a clone are unordered) in the WGP approach (van Oeveren *et al.* 2011). Second, though being analogous to optical mapping, RadMap is sequence based and is thus more convenient to be used in downstream applications (*e.g.*, a single unique tag is enough for anchoring a contig/scaffold). Third, RadMap does not rely on expensive specialized instruments or cell lines (as in optical mapping and the RH map), and is therefore more cost effective and accessible to laboratories focusing on diverse nonmodel species. Lastly, unlike other clone-based methods (*e.g.*, WGP, BAP), RadMap only creates subhaploid clone pools, and does not require the isolation and maintenance of individual fosmid/BAC clones, which can be a labor-intensive, time-consuming, and expensive procedure.

**Table 4:** Technical comparison between RadMap and other physical mapping methods

	RadMap	HAPPY map	RH map	WGP	Optical mapping
Map element	Restriction site	STS markers	STS markers	BAC clones	Restriction site
Map resolution (kb)	∼4	14	40	∼100–200	0.65
Clone format	Subhaploid clone pools	NA	NA	Individual clones	NA
Cell lines	NA	NA	Required	NA	NA
Marker system	2bRAD	STS	STS	AFLP	NA
Sequencing based	Yes	No	No	Yes	No
Specialized instrument	NA	NA	Linear accelerator	NA	OpGen Argus or BioNano Irys systems
Accessibility to a wide range of organisms	High	High	Low	High	High
Cost (including time and labor) for high-resolution map	Low	High	High	High	Medium
References	This study	Dear and Cook (1993); Konfortov *et al.* (2000)	Cox *et al.* (1990); Bach *et al.* (2012)	van Oeveren *et al.* (2011)	Teague *et al.* (2010); Levy-Sakin and Ebenstein (2013)

Our study shows that RadMap outperforms BioNano and Hi-C (the two most widely used scaffolding methods) when the contig N50 of input assembly is as low as 54 kb, suggesting that RadMap is well suited for scaffolding highly fragmented assemblies (which is common when initial assemblies are generated using short-read sequencing platforms). As for cost, producing a whole-genome restriction map for *Arabidopsis* by RadMap is ∼$1800, which includes the costs of the fosmid/2bRAD library preparation and Illumina sequencing of 100 subhaploid clone pools with 5 million reads for each pool. The commercial price for generation of a BioNano map for *Arabidopsis* is ∼$4000 (from DNA extraction to BioNano sequencing), while it is ∼$1900 for Hi-C library preparation and sequencing of a single *Arabidopsis* sample. Therefore, the cost for producing a whole-genome restriction map by RadMap is comparable to or cheaper than using the commercial services of Hi-C and BioNano. In addition, RadMap does not require a specialized instrument (compared with BioNano) and is relatively less technically demanding (compared to Hi-C), as fosmid/BAC library construction and RAD sequencing are both widely applied techniques. These advantages make RadMap an attractive method for genome scaffolding applications, especially when dealing with highly fragmented assemblies.

Population sequencing (POPSEQ) is analogous to RadMap but is based on a genetic-linkage mapping strategy (Mascher *et al.* 2013). Through whole-genome sequencing of a mapping population, POPSEQ allows for construction of an ultrahigh-density genetic map to facilitate the genome scaffolding process (Mascher *et al.* 2013). While the POPSEQ method is powerful, RadMap does have advantages over POPSEQ in some situations. First, RadMap is suited for genome mapping in species where making a controlled cross is impossible. Second, RadMap relies on *in-vitro* random chromosome breakage and segregation, whereas POPSEQ relies on *in-vivo* genetic recombination. POPSEQ may not be an appropriate choice when the target species has a highly uneven distribution of genetic recombination across the chromosome (*e.g.*, in an extreme case, recombination occurred only in ∼13% of the chromosome; see Sandhu and Gill 2002). Third, for genome scaffolding, gap size between contigs can be readily estimated by RadMap since it generates a physical map. The gap-size estimation is, however, difficult for POPSEQ as it generates a genetic map and “genetic” distance is more difficult to be converted to “physical” distance without prior knowledge of the recombination characteristics of target species.

To date, assembling highly heterozygous, polyploid, or largely expanded genomes remains very challenging. For mapping heterozygous genomes, we expect no major barrier for the RadMap approach. 2bRAD tags are very short (∼35 bp) so they should have very little chance of carrying many polymorphic loci. For example, containing one, two, and three polymorphic loci in a single tag corresponds to heterozygosity levels of 2.9, 5.7, and 8.6%, respectively, but the latter two levels are rarely seen in existing eukaryotic genomes (Leffler *et al.* 2012). Therefore, for most heterozygous genomes, the majority of 2bRAD tags in the genome should contain 0–1 polymorphic loci which would not affect the tag identity and mapping accuracy. In fact, RadMap may be a good choice for scaffolding heterozygous genomes as assembly of heterozygous genomes based on short-read platforms usually produces highly fragmented assemblies, and RadMap apparently outperforms other scaffolding methods (*e.g.*, BioNano and Hi-C) in such situations. However, for polyploid or largely expanded genomes, application of the RadMap approach may meet difficulties due to inefficient distinction of short tags from different genomic regions with high sequence similarity and/or insufficient clone sizes for forming effective linkage groups. In such situations, alternative methodologies may be more appropriate, *e.g.*, PacBio sequencing and assembly of fosmid/BAC dilution pools followed by Hi-C-based scaffolding.

### Genome scaffolding with high flexibility

Having a high-quality reference genome assembly for an organism is critical to the understanding of its biology and evolutionary relationship with other organisms. However, finishing a whole-genome assembly is often difficult (especially based on NGS platforms), leading to very few high-quality, chromosome-scale assemblies currently being available for large and complex genomes. Diverse strategies have been developed to boost the contiguity of WGS genome assemblies from short reads, with some competent at capturing midrange contiguity (*e.g.*, mate-pair sequencing and CPT-seq) and others at capturing chromosome-scale contiguity (*e.g.*, Hi-C and related methods). It is preferable for all levels of contiguity information to be collected from one approach. However, such “one-size-fits-all” approach remains yet underdeveloped. RadMap holds great potential to achieve this goal, as it can facilitate genome scaffolding with high flexibility in both genome connectivity and mapping resolution.

#### Choice of clone size:

RadMap can be coupled with different types of clone libraries to cover a wide range of genome connectivity, such as plasmid (∼2–4 kb), λ-phage (up to 25 kb), fosmid (∼35–40 kb), BAC (∼100–300 kb), yeast artificial chromosome (YAC) (up to 2 Mb). While we expect that fosmid/BAC libraries should be effective in most genome scaffolding applications, long-span libraries (*e.g.*, YAC) may be necessary when dealing with very large genomes.

#### Adjustment of marker density:

Apart from the restriction-enzyme replacement that is usually adopted in other GBS methods for marker density adjustment, the 2bRAD marker system also provides a convenient and reproducible way to fine tune marker density by means of selective adaptors (Wang *et al.* 2012; Jiao *et al.* 2014). For example, a wide range of representations can be potentially achieved in *Arabidopsis*, ranging from one-quarter of all sites (NNR overhang on both adaptors) to 1/256th of all sites (NGG overhangs on both adaptors). With this option, researchers can fine tune map resolution to meet specific purposes (*e.g.*, high marker density for fosmid/BAC libraries and low marker density for a long-span YAC library).

### Conclusion

We develop a sequencing-based HAPPY mapping approach, which provides an efficient and flexible tool for high-resolution physical mapping and genome scaffolding. We perform extensive analyses to validate the power and accuracy of our approach in both model and nonmodel species for generating high-quality restriction maps and enhancing the contiguity of WGS assemblies generated from short-read and long-read platforms. We envision that RadMap will become an integral part of the growing suite of scaffolding technologies for routine application in complex genome-sequencing projects to achieve chromosome-scale genome assemblies.

## Supplementary Material

Supplemental material is available online at www.genetics.org/lookup/suppl/doi:10.1534/genetics.117.200303/-/DC1.
