# Supplementary material for: Whole-Genome Restriction Mapping by “Subhaploid”-Based RAD Sequencing: An Efficient and Flexible Approach for Physical Mapping and Genome Scaffolding
Source: Genetics. 2017 May 2;206(3):1237–50. doi: 10.1534/genetics.117.200303 (PMC5500127; doi:10.1534/genetics.117.200303)
Supplement: Supplementary file 1 [file 1237FileS1.zip › AMMO2.0/doc/UserManual.docx]

**AMMO: an integrated package for whole-genome restriction mapping and genome scaffolding**

**(User Manual ver. 2.0)**

Jinzhuang Dou

Department of Marine Life Sciences

Ocean University of China

March 10, 2017

**Contents**

[1 Introduction 2](#_Toc476927610)

[2 Getting started 2](#_Toc476927611)

[2.1 Availability 2](#_Toc476927612)

[2.2 Installing AMMO 2](#_Toc476927613)

[2.3 Running AMMO 2](#_Toc476927614)

[3 Examples 3](#_Toc476927615)

[4 Input files 4](#_Toc476927616)

[4.1 PARAMETER_FILE (_.conf) 4](#_Toc476927617)

[4.2 INPUT_DIR (_.fasta) 4](#_Toc476927618)

[4.3 TEMPLATE_FILE (_.fasta) 5](#_Toc476927619)

[4.4 CTG_FILE (_.fasta) 5](#_Toc476927620)

[5 Usage options 5](#_Toc476927621)

[5.1 Main parameters 5](#_Toc476927622)

[5.2 Advanced parameters 6](#_Toc476927623)

[5.3 Command line arguments 6](#_Toc476927624)

[6 Output files 7](#_Toc476927625)

[6.1 _.log 7](#_Toc476927626)

[6.2 _.map 7](#_Toc476927627)

[6.3 _.fa 7](#_Toc476927628)

[6.4 _.link 8](#_Toc476927629)

[7 Version changes 8](#_Toc476927630)

[7.1 Version 1.0 (April 4^th^, 2015) 8](#_Toc476927631)

[7.2 Version 1.01 (June 5^th^, 2016) 8](#_Toc476927632)

[7.3 Version 2.0 (March 10^th^, 2017) 8](#_Toc476927633)

[8 Acknowledgements 9](#_Toc476927634)

[9 References 9](#_Toc476927635)

# 1 Introduction

AMMO is an integrated program that generates 2b-RAD [1]-based restriction maps for scaffolding draft genome assemblies produced by using short Illumina reads or long PacBio reads. The basic idea is to get the linkage information of contigs/ scaffolds from the pre-assemblies based on the HAPPY mapping experiment [2].

AMMO is advantageous in both experimental and computational aspects. First, AMMO combines the HAPPY mapping experiment with the high-throughput 2b-RAD sequencing technology, making the process of marker typing more cost-effective and less laborious in comparison with traditional HAPPY mapping approaches. Second, the hierarchical assembly algorithm implemented in AMMO is very efficient and can handle ultra-dense maps of up to 50,000~100,000 markers. Last, the bootstrap-basedsub-sampling strategy is adopted to reduce the linkage errors caused by noisy or incomplete mapping data.

Detailed description of the methodology and algorithms can be found in our paper titiled “Whole-genome restriction mapping by ‘subhaploid’-based RAD sequencing: an efficient and flexible approach for physical mapping and genome scaffolding”.

# 2 Getting started

## 2.1 Availability

The AMMO software package can be downloaded from the following webpage: <http://www2.ouc.edu.cn/mollusk/detailen.asp?id=752>. In the package, perl scripts for Linux (64-bit) operation systems are provided. The AMMO program uses the following bioinformatics tools: SOAP2 (version 2.1.1b) [3], Stacks (version 0.99997) [4], and MSTMap (version 1.0) [5].The executable programs of these tools have already been provided in the AMMO package, so users do not need to have these tools be pre-installed in your computer. Besides, user should also have the “bigmemory” “biganalytics”, “picante” and “amap” pacakges in your R session.

## 2.2Installing AMMO

Open a terminal and go to the directory where the AMMO.tar.gz file locates. Extract the file by typing tar –xvzfAMMO.tar.gz in the terminal. This will create a new directory called AMMO containing the executable perl scripts, required additional bioinformatics suits, and example datasets.

## 2.3Running AMMO

Remember the directory after extracting the .tar.gz file as $AMMO_PATH. Open a terminal and go to the directory that contains the raw sequencing datasets. Execute the program by typing:

*perl $AMMO_PATH/AMMO.pl –p parameterfile*

in which *–p* is the command line flag specifying the parameter file and *parameterfile* is the name the parameter file. If your *parameterfile* is not specified, you can turn to learn the command line arguments by typing:

*perl $AMMO_PATH/AMMO.pl –h*

For more about the *parameterfile* and command line arguments, see Section3 and Section 5.

# 3 Examples

This section provides anexemplar usage of the AMMO package based on the data in the folder named “*simdata*” (already included in the package). Please also refer to the next few sections for detailed information about input files (Section 4), usage options (Section 5), and output files (Section 6).

The“*simdata*” folder contains a parameterfile named “*example.conf*”, which specifies parameters for running AMMO on the simulated dataset, the file “*example.ctg*” is the pre-assembly generated using the software SOAP*denovo*, the file “*example.ref*” denotes the reference sequence derived from Chr.1 of *A. thaliana*. After decompressing the download package, enter the folder named“*simdata*”. The following command will use parameter values provided in the example parameterfile (shown at the end of this section).

*bash example.sh*

All the parameters required for AMMO are defined by “*example.conf*” file, which is similar to the one shown below. Each line specifies one parameter, followed by the parameter value (or followed by a “#” character if the parameter is undefined). Text after a “#” character in each line is treated as comment and will not be read by the program.

# This is a parameter file for AMMO v1.01.

# The entire line after a ‘#’ will be ignored.

###---Main Parameters----###

| INPUT_DIR | ./simtest | # no default value |
| --- | --- | --- |
| TEMPLATE_FILE | ./example.ctg | # no default value |
| GROUP_METHOD | 1 | #1 |
| CTG_FILE | ./example.ctg | # no default value |
| OUT_PREFIX | AMMO | # no default value |
| STAGE | markerType | #all |
| PARAMETER_FILE | ./example.conf | # no default value |
| SAMPLE_TIME | 20 | #20 |
| THRESHOLD | 0.6 | #0.6 |
| ITERATIVE_NUM | 10 | #10 |
| CPU_NUM | 10 | #1 |
| MIN_DEPTH | 2 | #2 |
| MIN_TYPING | 0.1 | #0.1 |
| MAX_TYPING | 0.9 | #0.9 |

###---Advanced Parameters----###

| SOAP_MATCH | 4 | # 4 |
| --- | --- | --- |
| USTACKS_M | 3 | # 3 |
| USTACKS_C | 2 | #1 |

###---Command line arguments ----###

| -p | PARAMETER_FILE |
| --- | --- |
| -d | INPUT_DIR |
| -t | TEMPLATE_FILE |
| -g | GROUP_METHOD |
| -c | CTG_FILE |
| -o | OUT_PREFIX |
| -S | STAGE |
| -s | SAMPLE_TIME |
| -T | THRESHOLD |
| -n | ITERATIVE_NUM |
| -P | CPU_NUM |
| -l | MIN_DEPTH |
| -u | MIN_TYPING |
| -v | MAX_TYPING |
| -m | SOAP_MATCH |
| -M | USTACKS_M |
| -C | USTACKS_C |
| -logfile | LOG_FILE |
| -debug | DEBUG_LEVLE |
| -h | HELP |

# 4 Input files

In this section, we describe four input files that are taken by AMMO package: INPUT_DIR, TEMPLATE_FILE, CTG_FILE and PARAMETER_FILE.

## 4.1 PARAMETER_FILE (_.conf)

The PARAMETER_FILE contains all parameters required for running AMMO. There are 17 parameters in the parameterfile, including 14 main parameters and 3 advanced parameters. Each parameter is followed by its assigned value, separated by white spaces/tabs. Text in the same line after a “#” character is treated as a comment and will not be read. For example, the following parameter specifications are equivalent in setting the parameter SAMPLE_TIME equal to 20:

| SAMPLE_TIME | 20 | #20 |
| --- | --- | --- |

An example PARAMETER_FILE is provided in Section 3. Four parameters do not have default values, which need to be explicitly defined by the users when in use, either in the parameterfile or in the command line (see Section 5). The other 13 parameters are pre-defined unless the user wants to use settings different from the default. Please refer to Section 5 for more information on these parameters.

## 4.2 INPUT_DIR (_.fasta)

The 2bRAD_FILE is the directory that contains sequence datasets for the 2b-RAD sequence dataset with the fasta format, and an example of sequencing file is provided below:

>Seq1

ACCTCCACGACGGCTCCGAACTTTT

## 4.3 TEMPLATE_FILE (_.fasta)

The TEMPLATE_FILE contains sequence datasets used to retain the reads that are derived from the targeted genome sequences. As DNA contamination (e.g. derived from fosmid vector and host bacteria) in HAPPY mapping experiments is usually unavoided, using TEMPLATE_FILE is necessary for removing these contaminated tags. The TEMPLATE_FILE can be contigs generated using *de novo* assemblers or short sequencing reads (e.g. Miseq PE300), for latter, the sequencing coverage of at least 10x is recommended.

## 4.4 CTG_FILE (_.fasta)

The CTG_FILE is the input file of AMMO for scaffolding. In principle, only contigs containing at least one BsaXI tags can be anchored using 2b-RAD map. Since the average distance between adjacent BsaXI tags being 4kb theoretically, the average size of contigs longer than 10kb is recommended. As mentioned above, CTG_FILE can also be implemented as a template sequence to filter the foreign tags.

# 5 Usage options

AMMO has 17 parameters that users can set in the PARAMETER_FILE, including 14 main parameters that are required for running AMMO and 3 advanced parameters for specialized options. Among the 14 main parameters, 4 parameters regarding the input data file need to be explicitly defined by users. The other 10 parameters and the 3 advance parametersarepre-defined as default values.

## 5.1 Main parameters

| INPUT_DIR | str | The directory that contains the 2b-RAD sequence dataset in the fasta format. |
| --- | --- | --- |
| PARAMETER_FILE | str | The file contains all parameters required for running AMMO. |
| TEMPLATE_FILE | str | The file contains sequence datasets that are derived from the targeted genome sequences. |
| CTG_FILE | str | The file contains the input contigs for AMMO scaffolding. |
| OUT_PREFIX | str | The prefix that will be added to the file names of output results. The default value is “AMMO”. |
| STAGE | str | This parameter allows to run AMMO.pl starting from specified steps, including all, templateFilter, markerType, markerGroup, markerSort, and GapFill. (default all) |
| GROUP_METHOD | int | Whether build the physical map composed of 2b-RAD tags or scaffold the pre-assemblies. Set this parameter as 0 for the former, and as 1 for the latter. (default 1) |
| SAMPLE_TIME | int | The maximal sampling times required for getting marker linkage information. (default 20) |
| THRESHOLD | float | The minimal time of sampling required to build a link between pair-wise markers during the sampling process. (default 0.6) |
| ITERATIVE_NUM | int | The maximal iterative times allowed for getting marker linkage information. (default 10) |
| CPU_NUM | int | Number of processors used during the sampling process. (default 1) |
| MIN_DEPTH | int | Minimal sequencing depth allowed for marker typing. (default 2) |
| MIN_TYPING | int | The minimal percentage of positive typing within the mapping panel, a threshold for discarding the 2b-RAD tags resulting from sequencing error. (default 0.1) |
| MAX_TYPING | int | The maximal percentage of positive typing within the mapping panel, a threshold for removing the 2b-RAD tags derived from repetitive genomic regions. (default 0.9) |

## 5.2 Advanced parameters

| SOAP_MATCH | int | Match mode for each read or the seed part of read mapping using SOAP2, which shouldn't contain more than 2 mismaches, (default 4).  0: exact match only  1: 1 mismatch match only  2: 2 mismatch match only  4: find the best hits |
| --- | --- | --- |
| USTACKS_M | int | Minimum depth of coverage required to create a stack (default 3) |
| USTACKS_c | int | Maximum distance (in nucleotides) allowed between stacks (default 2). |

## 5.3 Command line arguments

The command line flags provide the user an option to enter information from the command line. All command line arguments will overwrite values specified in the PARAMETER_FILE. If a parameter is specified with an invalid value in the PARAMETER_FILE but a valid value in the command line, the program will return a warning message and still execute correctly by taking the value from the command line. However, if a parameter value in the command line is not valid, the program will exit with an error message. If a command line flag is specified, it must be followed by a space and then the parameter value. Different command line flags can appear in any order. Most command line arguments are optional except for the four input files mentioned in Section 4. One example is shown as following:

*perl $AMMO_PATH/AMMO.pl –p example.conf*

*–logfile* is the command line flag specifying the log file that records the error message for AMMO, however, you must set the debug level 10 to make this option valid. Or you can run AMMO step by step as following:

*perl $AMMO_PATH/AMMO.pl –p example.conf -S templateFilter*

*perl $AMMO_PATH/AMMO.pl –p example.conf -S markerType*

*perl $AMMO_PATH/AMMO.pl –p example.conf -S markerGroup*

*perl$AMMO_PATH/AMMO.pl –p example.conf-S markerSort-p 10 -s 20 -t 100 -c 0.6*

*–g 2bRADtyping.ctg.type -p 10 -s 20 -t 100 -c 0.6 -a 1 -b 5 -l 0*

*perl $AMMO_PATH/AMMO.pl –p example.conf-p example.conf -S GapFill*

# 6 Output files

All output files will be saved in the current directory and will start with the parameter value of OUT_PREFIX. These files are described as below.

## 6.1 _.log

The terminal outputs are used to monitor and record the process when running AMMO. It starts with all parameter values specified, and reports the progress of the program step by step. The log file is identical to the terminal outputs.

## 6.2 _.map

The map file which is self-explanatory and easy to understand records. The first line is the header line. Starting from the second line, each line represents one 2b-RAD tag. The first column is the marker IDs and the following three columns represent the groupIDs, scaffold/contig ID estimated genetic distance along the scaffold. The file is tab-delimited. Below is an illustration of the format of the .map file.

| Consensus1 | lg1 | Contig1 | 0.000 |
| --- | --- | --- | --- |
| Consensus2 | lg1 | Contig2 | 4.009 |
| Consensus3 | lg1 | Contig6 | 7.174 |
| Consensus4 | lg2 | Contig3 | 0.000 |
| Consensus5 | lg2 | Contig3 | 15.000 |

## 6.3 _.fa

The _.fa file contains the final scaffold sequences obtained using AMMO with CTG_FILE as an input.

## 6.4 _.link

The_.linkfile contains the link information for pairs of scaffold. Starting from the first line, each line represents the pair-wise link of scaffolds whose link times over the threshold setting by parameter THRESHOLD. The first column is the marker IDs and, the second column is the times that this pairs of scaffolds are adjacent to each other during the whole sampling process, the third column is the estimated genetic distance for this pairs of scaffolds, the four column denotes the corresponding scaffold/contig IDs they belong to, and the last column means the groupID they finally locate in. The file is tab-delimited. Below is an illustration of the format of the _.link file.

| (Consensus1Consensus2) | 25 | 0.251 | (tig311 tig298) | Lg1 |
| --- | --- | --- | --- | --- |
| (Consensus5 Consensus7) | 18 | 0.386 | (tig301 tig2) | Lg2 |
| (Consensus4 Consensus2) | 25 | 0.211 | (tig1 tig298) | Lg1 |
| (Consensus8 Consensus9) | 11 | 0.316 | (tig301 tig2) | Lg5 |
| (Consensus6 Consensus3) | 15 | 0.101 | (tig11 tig98) | Lg4 |

# 7 Version changes

Changes from previous versions of this software are noted here.

## 7.1 Version 1.0(April 4^th^, 2015)

- Initial release of the AMMO package.

## 7.2 Version 1.01(June 5^th^ ,2016)

- The module of estimating internal gap sizes has been provided.

- Added new parameter“MIN_DEPTH”to allow users to determine the minimal

sequencing depth for marker typing has been provided.

## 7.3 Version 2.0(March 10^th^ ,2017)

- Changed the AMMO program to be much more memory efficient by using the

functions provided in the R package “bigmemory”. Now, the new version can

handle ultra-dense maps of up to ~1M markers with efficient memory usage.

- Updated MSTMap (Wu et al., 2008) software to the multi-threading feature to

allow user to obtain optimal marker order much faster than previous version.

- Updated the software manual.

# 8 Acknowledgements

# 9 References

[1] Wang S, Meyer E, McKay J K, et al. 2b-RAD: a simple and flexible method for genome-wide genotyping. Nature Methods, 2012, 9(8): 808-810.

[2] Dear P H, Cook P R. Happy mapping: linkage mapping using a physical analogue of meiosis. Nucleic Acids Research, 1993, 21(1): 13-20.

[3] Li R, Yu C, Li Y, et al. SOAP2: an improved ultrafast tool for short read alignment. Bioinformatics, 2009, 25(15): 1966-1967.

[4] Catchen J M, Amores A, Hohenlohe P, et al. Stacks: building and genotyping loci de novo from short-read sequences. G3: Genes, Genomes, Genetics, 2011, 1(3): 171-182.

[5] Wu Y, Bhat P R, Close T J, et al. Efficient and accurate construction of genetic linkage maps from the minimum spanning tree of a graph. PLoS Genetics, 2008, 4(10): e1000212.
