# Supplementary material for: Whole-Genome Restriction Mapping by “Subhaploid”-Based RAD Sequencing: An Efficient and Flexible Approach for Physical Mapping and Genome Scaffolding
Source: Genetics. 2017 May 2;206(3):1237–50. doi: 10.1534/genetics.117.200303 (PMC5500127; doi:10.1534/genetics.117.200303)
Supplement: Supplementary file 2 [file 1237FileS2.pdf]

Supplementary Materials

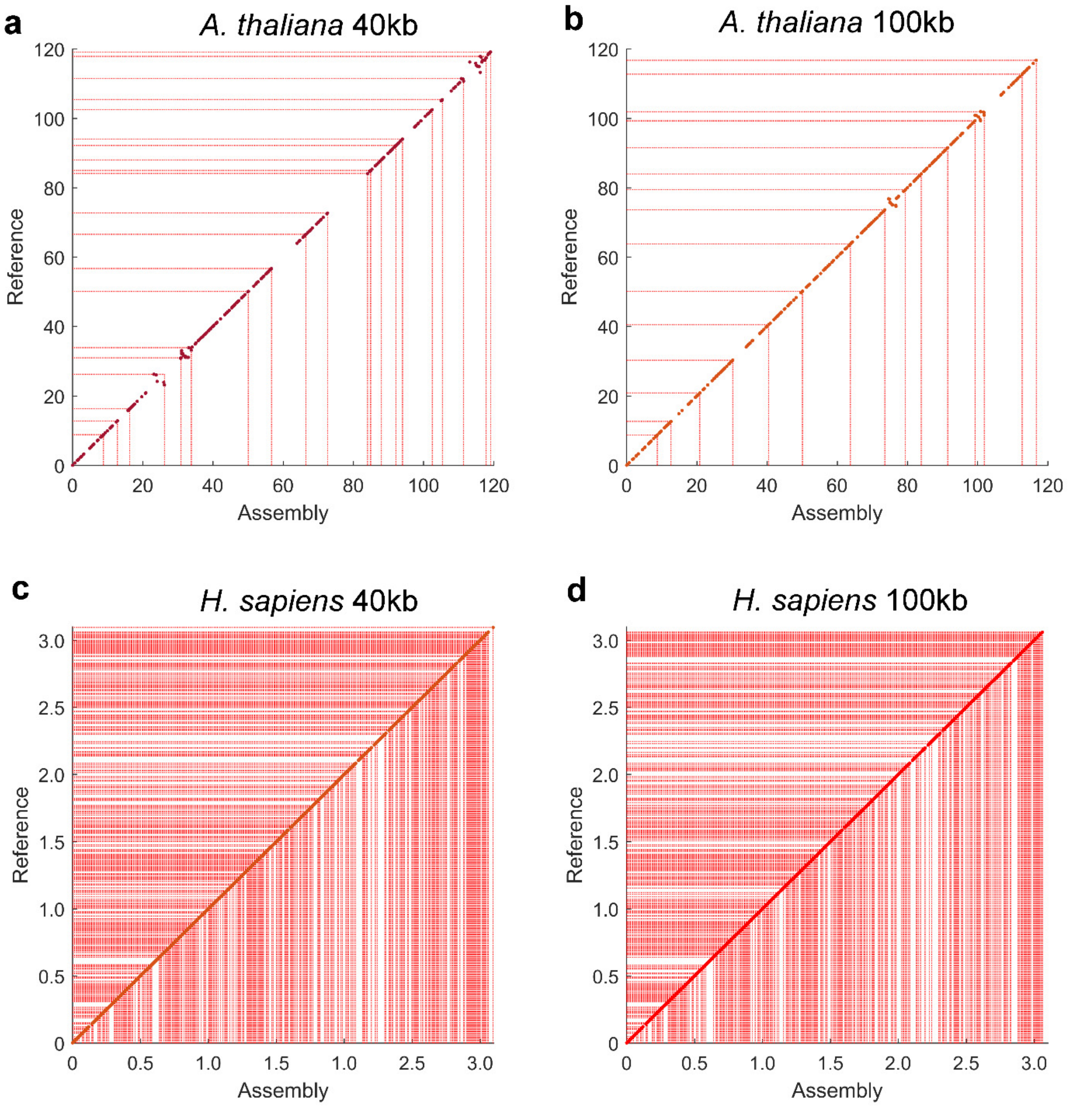

Figure S1. Dot-plot comparisons showing that restriction maps generated using simulation datasets are highly consistent with the reference genomes. (a) *A. thaliana* 40kb dataset; (b) *A. thaliana* 100kb dataset; (c) human 40kb dataset; (d) human 100kb dataset. For each assembled map, break points are marked with red lines.

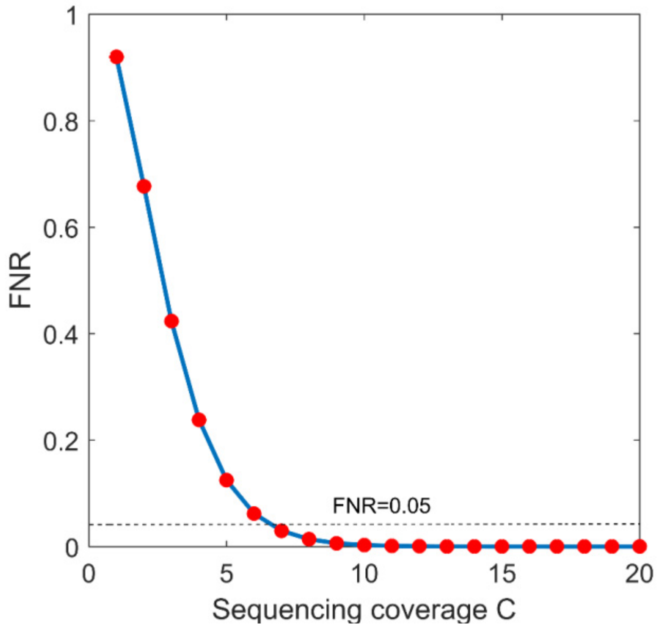

Figure S2. The false negative rate (FNR) as the function of average sequencing coverage, showing that the average sequencing coverage of RadMap should theoretically be no less than 7x in order to ensure a low false negative rate (FNR<0.05) of marker genotyping.

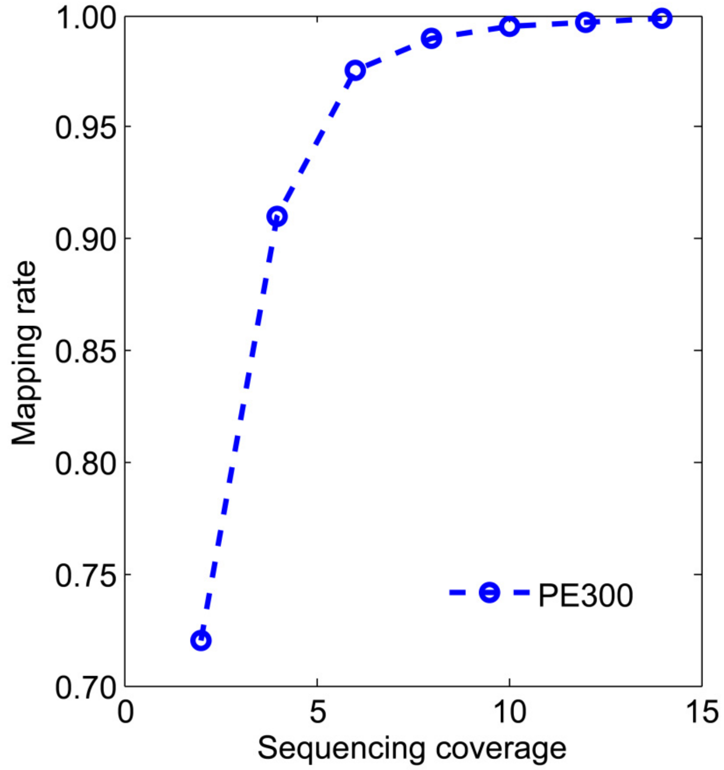

Figure S3. The mapping rate of *Bsa*XI tags as a function of WGS sequencing coverage, showing that 10x MiSeq PE300 reads is sufficient to capture nearly all *Bsa*XI tags (99.7%) from the complete genome of *A. thaliana*.

Table S1. Overview of the *Bsa*XI tags *in silico* extracted from the genomes of *A. thaliana* and *H. sapiens*.

	A. thaliana		*H. sapiens*	
Clone size	40 kb	100 kb	40 kb	100 kb
Total number of *Bsa*XI tags	39,678	39,678	1,165,976	1,165,976
Total number of unique tags[a]	35,618	35,618	1,118,736	1,118,736
Ave. distance between two unique tags (bp)	3,344	3,344	3,041	3,041
Max. distance between two unique tags (bp)	228,876	228,876	404,226	404,226
Min. distance between two unique tags (bp)	2	2	0	0
Standard deviation (bp) of tag distances	4,452	4,452	8,367	8,367

[a] unique tags refer to those that can map to a unique genomic location.

Table S2. Summary of hierarchical restriction mapping for simulation datasets.

	A. thaliana		*H. sapiens*	
	No. of groups (40kb)	No. of groups (100kb)	No. of groups (40kb)	No. of groups (100kb)
Step1	409	176	1,368	1,314
Step2	151	80	1,303	1,262
Step3	75	40	1,074	1,150
Step4	60	31	975	970
Step5	49	28	704	690
Step6	49	27	596	567
Step7	44	25	534	475
Step8	42	24	493	418
Step9	40	20	407	361
N50 (Mb)	4.1	12.7	9.7	11.0
N90 (Mb)	0.8	4.9	2.7	2.8
Coverage	98.4%	98.5%	99.2%	99.4%
Accuracy[a]	99.7%	99.9%	99.9%	99.9%

[a] Calculated according to Kendall's statistic.

Table S3. Summary of 2b-RAD sequencing of 164 “subhaploid” clone pools in *A. thaliana*.

Clone Pool ID	No. of reads	Depth (per BsaXI tag)	No. of estimated clones	Haploid genome coverage
1	8,798,940	45.81	778	0.25
2	8,598,616	46.40	752	0.25
3	8,888,729	43.89	552	0.17
4	7,878,066	44.34	538	0.16
5	7,745,652	51.31	542	0.16
6	9,012,666	51.12	568	0.17
7	6,644,057	51.01	520	0.15
8	9,262,581	42.74	591	0.18
9	8,838,650	44.50	517	0.15
10	8,416,638	50.28	594	0.17
11	7,726,545	44.01	553	0.17
12	8,320,462	44.20	531	0.16
13	7,878,340	47.23	545	0.15
14	8,061,126	48.85	538	0.17
15	8,054,874	44.97	625	0.19
16	9,153,445	43.54	594	0.18
17	8,554,666	46.97	579	0.17
18	9,779,217	44.20	603	0.19
19	10,467,344	44.36	578	0.18
20	9,877,001	47.10	580	0.18
21	9,340,311	46.17	589	0.18
22	8,724,105	44.53	602	0.17
23	9,634,225	50.85	617	0.18
24	8,156,842	44.57	559	0.17
25	7,847,942	49.81	776	0.24
26	9,170,107	47.69	596	0.19
27	10,228,036	50.08	560	0.17
28	9,178,701	50.51	574	0.17
29	8,881,887	44.71	578	0.17
30	9,294,126	48.76	604	0.18
31	10,002,171	47.68	593	0.18
32	10,160,853	44.56	550	0.17
33	9,529,320	46.27	565	0.17
34	10,087,763	44.56	574	0.18
35	11,321,672	45.07	554	0.17
36	9,715,208	51.03	530	0.16
37	10,063,069	44.48	664	0.19
38	9,634,494	46.57	601	0.18
39	8,948,585	44.03	572	0.16
40	9,846,109	48.56	537	0.17
41	11,602,099	44.06	579	0.17
42	11,322,493	47.48	595	0.18
43	11,776,939	47.74	746	0.25
44	9,058,792	46.69	704	0.25
45	8,709,648	48.46	750	0.27
46	9,069,226	51.65	555	0.17
47	9,893,385	49.71	628	0.19
48	9,365,082	44.53	614	0.19
49	9,441,565	42.80	580	0.18
50	8,948,328	42.86	662	0.22
51	9,264,343	44.32	684	0.21
52	7,262,044	50.64	725	0.22

53	8,143,980	48.42	659	0.19
54	7,036,271	46.19	784	0.22
55	6,530,909	42.42	553	0.16
56	8,498,443	49.46	538	0.16
57	8,266,795	47.61	731	0.23
58	8,773,181	50.79	600	0.17
59	7,918,517	43.63	690	0.21
60	9,235,838	42.92	607	0.18
61	8,599,452	50.58	641	0.18
62	8,736,217	43.86	610	0.19
63	8,542,532	49.67	587	0.18
64	8,791,722	42.83	667	0.20
65	9,553,228	42.54	601	0.18
66	7,676,836	42.80	559	0.17
67	8,236,655	51.24	744	0.25
68	8,440,741	48.68	665	0.21
69	8,780,652	48.75	655	0.20
70	10,448,499	50.76	685	0.23
71	10,128,486	51.34	551	0.16
72	9,859,292	43.69	578	0.17
73	10,147,356	46.10	575	0.18
74	9,568,286	44.13	578	0.17
75	8,161,269	42.96	608	0.19
76	8,877,768	42.89	564	0.15
77	10,205,035	46.34	566	0.17
78	9,261,948	43.92	591	0.18
79	8,620,603	49.89	829	0.32
80	9,509,822	47.48	757	0.26
81	9,039,585	43.14	538	0.16
82	10,135,141	45.09	685	0.23
83	9,624,074	44.29	530	0.15
84	8,375,640	44.77	718	0.26
85	7,932,895	47.03	587	0.17
86	8,386,775	49.28	662	0.21
87	5,793,920	43.86	646	0.22
88	7,611,486	46.79	713	0.22
89	8,052,909	48.83	695	0.23
90	8,317,600	46.31	693	0.25
91	9,229,832	42.85	738	0.26
92	9,228,943	42.48	703	0.23
93	8,715,776	43.72	705	0.25
94	8,831,001	45.74	710	0.25
95	9,999,178	50.39	698	0.24
96	8,904,443	43.33	664	0.21
97	8,362,104	45.86	865	0.33
98	9,088,621	47.26	719	0.23
99	8,474,315	50.42	684	0.22
100	7,578,404	44.29	806	0.28
101	9,323,251	46.62	689	0.23
102	9,467,590	44.92	704	0.24
103	9,559,497	47.79	677	0.23
104	10,062,353	49.21	718	0.25
105	9,157,928	46.20	849	0.32
106	10,057,412	42.64	655	0.21
107	9,353,350	46.38	655	0.22
108	9,447,200	48.09	717	0.25
109	8,989,967	44.12	643	0.21

110	8,304,319	49.81	688	0.24
111	8,994,787	48.89	637	0.21
112	13,579,917	51.21	722	0.26
113	12,205,953	49.42	790	0.27
114	13,736,878	50.19	695	0.24
115	13,607,838	49.41	765	0.26
116	9,112,926	49.04	694	0.24
117	13,612,130	48.62	681	0.23
118	8,356,257	47.94	924	0.35
119	8,552,146	44.49	667	0.25
120	7,564,509	47.77	675	0.23
121	7,363,811	45.86	761	0.26
122	7,881,337	49.13	693	0.23
123	7,621,375	45.32	723	0.25
124	7,154,029	47.95	711	0.24
125	8,650,434	43.72	718	0.25
126	8,357,194	47.37	658	0.23
127	8,529,860	50.23	731	0.24
128	7,859,845	46.83	720	0.26
129	8,286,040	48.25	702	0.25
130	7,446,962	43.43	813	0.31
131	8,009,365	43.25	809	0.27
132	8,796,193	50.76	678	0.24
133	8,141,142	44.16	750	0.24
134	8,908,351	45.77	670	0.22
135	9,255,335	46.51	735	0.25
136	9,038,673	48.82	682	0.23
137	8,533,837	47.89	721	0.23
138	7,953,903	49.83	699	0.24
139	8,648,765	44.71	821	0.28
140	8,805,351	44.09	789	0.28
141	9,391,814	52.30	693	0.24
142	9,628,395	43.21	829	0.28
143	10,179,407	49.93	724	0.23
144	9,771,412	42.80	660	0.23
145	9,647,385	48.88	694	0.24
146	9,483,989	48.33	708	0.25
147	9,756,173	48.68	685	0.24
148	9,064,523	47.58	701	0.24
149	7,928,423	48.52	700	0.23
150	9,618,805	51.48	656	0.23
151	9,712,825	48.41	782	0.27
152	10,011,232	49.68	697	0.24
153	8,749,287	47.74	728	0.25
154	8,886,614	47.01	734	0.25
155	8,361,511	48.18	731	0.24
156	8,965,584	51.67	761	0.25
157	8,927,606	50.22	728	0.25
158	7,718,804	46.49	814	0.28
159	7,166,751	42.76	804	0.27
160	7,785,611	50.41	723	0.26
161	7,643,381	45.90	838	0.27
162	7,483,375	47.33	701	0.24
163	7,886,703	49.52	671	0.23
164	8,167,231	47.39	672	0.23

Table S4. Summary of WGS datasets used for *de novo* assembly in this study.

	MiSeq PE300	PacBio1	PacBio2[a]
Avg. read length (bp)	300	5,479	14,711
Total no. of reads	10,074,732	109,327	40,445
Genome coverage	25x	5x	5x

[a] This dataset was retrieved from the website of Pacific Biosciences (http://www.pacb.com/blog/new-data-release-arabidopsis-assembly/).

Table S5. Summary of WGS assemblies generated using four *de novo* assemblers.

Dataset	#Contigs	N50 (kb)	N90 (kb)	Coverage (Mb)	Software
MiSeq PE300	4,502	54.1	12.2	115.7 (96.6%)	Celera
	12,247	24.5	4.3	107.3 (90.2%)	SOAPdenovo2
	16,157	16.3	3.2	111.1 (93.3%)	ABySS
	67,752	37.2	0.5	164.5 (138.2%)	SPAde
